# Antibodies against MERS Coronavirus in Dromedary Camels, Kenya, 1992–2013

**DOI:** 10.3201/eid2008.140596

**Published:** 2014-08

**Authors:** Victor M. Corman, Joerg Jores, Benjamin Meyer, Mario Younan, Anne Liljander, Mohammed Y. Said, Ilona Gluecks, Erik Lattwein, Berend-Jan Bosch, Jan Felix Drexler, Set Bornstein, Christian Drosten, Marcel A. Müller

**Affiliations:** University of Bonn Medical Centre, Bonn, Germany (V.M. Corman, B. Meyer, J.F. Drexler, C. Drosten, M.A. Müller);; International Livestock Research Institute, Nairobi, Kenya (J. Jores, A. Liljander, M.Y. Said);; Vétérinaires Sans Frontières Germany, Nairobi (M. Younan);; Vétérinaires Sans Frontières Suisse, Nairobi (I. Gluecks);; EUROIMMUN AG, Lübeck, Germany (E. Lattwein);; Utrecht University, Utrecht, the Netherlands (B.-J. Bosch);; National Veterinary Institute, Uppsala, Sweden (S. Bornstein)

**Keywords:** Middle East respiratory syndrome coronavirus, MERS-CoV, viruses, coronavirus, dromedary camels, reservoir, antibodies, seroprevalence, zoonoses, Kenya

## Abstract

Dromedary camels are a putative source for human infections with Middle East respiratory syndrome coronavirus. We showed that camels sampled in different regions in Kenya during 1992–2013 have antibodies against this virus. High densities of camel populations correlated with increased seropositivity and might be a factor in predicting long-term virus maintenance.

Middle East respiratory syndrome coronavirus (MERS-CoV) was discovered in a patient from Saudi Arabia in 2012 and has since caused ≥250 human infections and 93 deaths (*1*). The evolutionary origins of MERS-CoV and related viral species belonging to the genus *Betacoronavirus* clade C were attributed to insectivorous bats in Europe and Africa (*2**–**4*). Seroprevalence studies of livestock from diverse species showed that dromedary camels from Oman, Saudi Arabia, the United Arab Emirates, Jordan, Qatar, Spain, and Egypt harbored antibodies against MERS-CoV antigens (*5**–**8*). Direct evidence for MERS-CoV infection in camels has been found in Qatar, Saudi Arabia, and Egypt. Close similarity of camel-associated and human-associated MERS-CoV sequences suggests that camels are sources of infection for humans and might constitute a zoonotic animal reservoir (*5**,**9**,**10*). Where and when the putative introduction of MERS-CoV into camel populations took place and how the virus is maintained in camel populations remains obscure.

Most livestock camels slaughtered in the Arabian Peninsula and in Egypt are imported from the Greater Horn of Africa, in particular Ethiopia, Somalia, Sudan, and Kenya (*11**,**12*). We investigated MERS-CoV antibody levels and distribution patterns in farmed and nomadic camels from Kenya.

## The Study

Samples were obtained from 774 dromedary camels in 3 regions in Kenya (Northeastern, Eastern, and Rift Valley [former administrative provinces]) and 7 counties (Mandera, Wajir, Isiolo, Marsabit, Laikipia, Turkana, and Baringo) during 1992–2013 (Figure). Blood samples were obtained from farmed or nomadic camels by jugular vein puncture. Serum samples originated from the archives of the International Livestock and Research Institute (ILRI) (Nairobi, Kenya). Ethical clearance for collection was part of the agreement between the Government of Kenya and ILRI, which provided ILRI with approval to broadly investigate livestock disease in Kenya.

**Figure:**
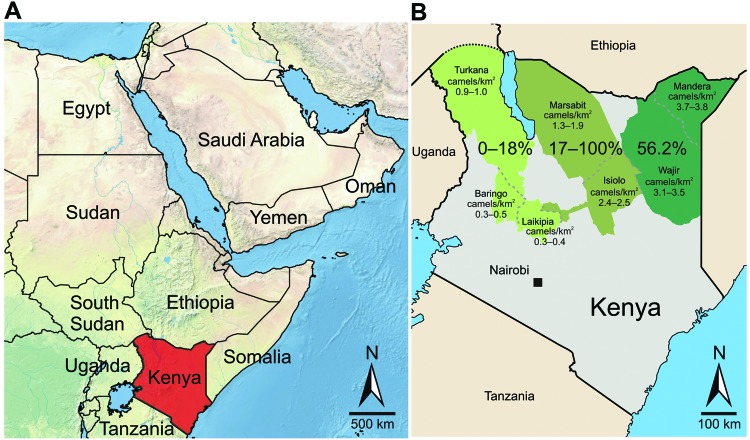
Greater Horn of Africa and Kenya. A) Arabian Peninsula and neighboring countries in the Greater Horn of Africa. B) Detailed map of Kenya showing sampling sites in 7 counties (Turkana, Baringo, Laikipia, Marsabit, Isiolo, Mandera, and Wajir) for Middle East respiratory syndrome coronavirus (MERS-CoV). Counties were assigned to 3 regions named after the former administrative provinces of Rift Valley, Eastern, and Northeastern (left to right). The 3 sampling regions are indicated in shades of green and other counties are indicated in gray. Percentages of camels positive for antibodies against MERS-CoV are shown with the density of camels (individuals/km^2^) for the analyzed regions during 2 periods (1991–2000 and 2000–2013). Serosurveys were performed during 1992–2013. Camel population numbers were determined for 1991–2000 and 2000–2013. Maps were created by using data from http://www.naturalearthdata.com.

All serum samples were tested for MERS-CoV antibodies by using a recombinant MERS-CoV spike protein subunit 1–based ELISA (rELISA) as described (*13*). Serum samples were used at a 1:100 dilution, which had been shown to be optimal for screening (*13*). A positive serum sample from recent studies (*6**,**13*) was used as a reference in all experiments. We used the assay-specific cutoff (optical density ratio 0.3) that had been validated in a previous study of camel serum samples (*13*). A total of 228 (29.5%) of 774 dromedary camels were rated MERS-CoV positive by the rELISA (Table 1). All 228 rELISA-positive serum samples from these 228 camels were subsequently tested at a 1:40 dilution by using an established recombinant immunofluorescence assay and Vero cells expressing MERS-CoV spike protein (*6*). This confirmatory assay showed that 213 (93.4%) of 228 rELISA-positive serum samples had MERS-CoV antibodies (Table 1).

**Table 1:** Analysis for MERS-CoV in serum samples of dromedary camel from 3 regions in Kenya, 1992–2013*

Region	County†	Husbandry/ management	Year	No. samples	No. rELISA positive samples (%)	No. rIFA positive samples/rELISA positive samples (%)
Northeastern	Mandera/ Wajir‡	Nomadic§	2008	162	91 (56.2)	86/91 (94.5)
Eastern	Isiolo	Nomadic§	1998	12	2 (16.7)	1/2 (50.0)
	Marsabit	Nomadic§	1999	41	32 (78.0)	28/32 (87.5)
	Variable	Nomadic§	2000	73	39 (53.4)	38/39 (97.4)
	Marsabit	Nomadic§	2008	21	12 (57.1)	12/12 (100.0)
	Marsabit	Nomadic§	2013	7	7 (100.0)	7/7 (100.0)
Rift Valley	Laikipia¶	Ranch#	1992	22	1 (4.5)	0/1 (0.0)
	Laikipia	Ranch#	1996	37	2 (5.4)	2/2 (100.0)
	Laikipia	Ranch#	1998	50	0 (0.0)	ND
	Laikipia	Ranch#	1999	175	32 (18.3)	30/32 (93.8)
	Turkana	Nomadic**	1999	50	7 (14.0)	6/7 (85.7)
	Laikipia	Ranch#	2000	56	2 (3.6)	2/2 (100.0)
	Baringo	Research center††	2007	28	0 (0.0)	ND
	Laikipia	Ranch	2013	40	1 (2.5)	1/1 (100.0)
Total				774	228 (29.5)	213/228 (93.4)

*MERS-CoV, Middle East respiratory syndrome coronavirus; rELISA, recombinant ELISA for MERS-CoV subunit 1 spike protein (serum samples were tested at a dilution of 1:100); rIFA, recombinant immunofluorescence assay for MERS-CoV spike protein expression in Vero cells (serum samples were tested at a dilution of 1:40; ND, not done. †Designated county refers to place of sampling or location in which camels were primarily located. ‡Data was merged because both counties had comparable antibody levels. §High density of camels and regular contact between herds, including exchange of animals between herds in relation to lactation and reproduction status. ¶Formerly from Pakistan. #Low density of camels and only sporadic contact between herds, with introduction of new animals only by purchase or livestock raiding, or restocking of camels. **Low density of camels but more frequent contact between herds than on ranches This includes encounters at waterholes and night enclosures, as well as sharing of pastures with daily to weekly contact between herds. ††Isolated herd that originated in Wajir but was kept under quarantine-like isolation conditions for experimental work since 1998.

As a final step, antibody specificity was confirmed by using a highly specific MERS-CoV microneutralization assay as described (*6*). All 228 rELISA-positive serum samples were tested at a starting dilution of 1:80 and an ending dilution of 1:800 to identify animals with high neutralization titers. A total of 119 (52.2%) 228 rELISA-positive serum samples had MERS-CoV neutralizing antibody titers (range 1:80–1:800) and 14 (6.1%) of 228 had high (>1:800) titers. The highly reactive camel serum samples originated from 3 counties (Wajir, Mandera, and Marsabit) in 2 regions (Northeastern and Eastern). The highest determined endpoint titer was 1:5,120.

Dromedary camels that had MERS-CoV antibodies were present at all sampling sites and during the 20-year sampling period (Table 1; Figure). With the exception of 1 county, seroprevalence was generally higher in the Northeastern and Eastern regions (range 53.4%–100%) than in the northern Rift Valley region (range 0%–17.5%). 

Serum samples from 28 dromedary camels from Wajir County that had been held at a research center in isolation conditions since 1998 were negative for MERV-CoV antibodies. To further confirm the observed seropositivity gradient, we compared those 129 camel serum samples with those that were obtained in the same year (2000) but at 2 locations (Eastern and northwestern Rift Valley regions). Antibody levels of nomadic dromedary camels from the Eastern region were significantly higher than those for farmed animals from the Rift Valley (corrected χ^2^ 34.1, p<0.005) (Table 2). Adult animals in both regions had a 7%–10% higher seroprevalence than juvenile animals, which is consistent with results of a previous study (*6*).

**Table 2:** Antibodies against MERS-CoV in dromedary camels in 2 regions of Kenya, 2000*

Region	County†	Husbandry	Sex	Age	No. samples positive by rELISA/no. tested (%)
Eastern	Marsabit	Nomadic‡	F/M	A	24/42 (57.1)
			F/M	J	15/31 (48.4)
Subtotal					39/73 (53.4)
Rift Valley	Laikipia	Ranch§	F/M	A	2/28 (7.1)
			F/M	J	0/28 (0)
Subtotal					2/56 (3.6)
Total					41/129 (31.8)

*MERS-CoV, Middle East respiratory syndrome coronavirus; rELISA, recombinant ELISA; A, adult; J, juvenile. †Designated county refers to place of sampling or location in which camels were primarily located. ‡Frequent herd contacts. §Sporadic herd contacts.

Because virus transmission might be influenced by population density, we attempted to correlate seroprevalence with dromedary camel population density across different regions. Data for dromedary camel density (Technical Appendix) were calculated on the basis of livestock counts conducted by the Department of Resource Surveys and Remote Sensing as part of an ongoing Kenya-wide rangeland monitoring program (*14*). Increased seroprevalence showed a significant correlation (Spearman rank correlation coefficient 0.715, p<0.005) with higher densities of dromedary camel populations in the Northeastern region and the northern part of the Eastern region (range 0.73–2.9 animals/km^2^) than in the Rift Valley region (0.58–0.6 animals/km^2^) (Figure; Technical Appendix).

## Conclusions

The present study showed that dromedary camels from Kenya have antibodies against MERS-CoV, which complements the current finding that MERS-CoV is a common pathogen in dromedary camel populations (*5**,**6**,**8**,**9**,**13*). Our finding of MERS-CoV antibodies in dromedary camels as early as 1992 is consistent with findings of a recent report from Saudi Arabia, which suggested that MERS-CoV has been circulating in dromedary camels for ≥20 years (*5*).

To project and potentially control virus spread, the public health community must understand factors determining virus maintenance. Our group and others have demonstrated that young dromedary camels have lower seroprevalences and are more likely to carry infectious virus (*5**,**6*). Similar observations have been made for coronaviruses in their original chiropteran hosts wherein strong virus amplification occurred soon after the time of parturition (*15*). Young, immunologically naive animals may thus facilitate virus amplification in dromedary camel populations.

We also demonstrated that dromedary camel population density shows a positive correlation with MERS-CoV seropositivity, which suggests efficient MERS-CoV maintenance or spread if herd density is high. Different types of animal husbandry in the Northeastern and Eastern regions of Kenya might be better predictors of virus transmission among camels. Dromedary camels in this area are often nomadic following rainfall patterns, and are taken across borders into neighboring countries, such as Ethiopia, for trade purposes (*13*). The observed increase in seropositivity from the Western region to the Northeastern and Eastern regions could be attributed to increased animal-to-animal contact in cross-border dromedary camel metapopulations.

Conversely, dromedary camels that originated in the Northeastern region but had been held in isolation since 1998 were negative for MERS-CoV antibodies, which is consistent with absence of antibodies in dromedary camels bred in isolation in Dubai (*6*). The combination of nomadic husbandry for a large population and presence of young virus-susceptible animals might facilitate virus maintenance. However, our retrospective study with archived samples could not assess hypotheses for each of the individual variables to determine their relative and absolute degrees of influence on virus circulation.

Because exportation of dromedary camels is largely unidirectional from eastern Africa into the Arabian Peninsula (*11*), our findings might facilitate the search for more ancestral MERS-CoV variants to clarify the natural history of acquisition of MERS-CoV by dromedary camels and its putative transmission to humans. Our recent finding of a MERS-CoV ancestor in bats from South Africa (*3*) highlights the need for wider investigations of viral reservoirs. The fact that no human MERS cases have been observed in eastern Africa could indicate less transmissibility of viruses in regional lineages or lack of detection and reporting of cases. Serosurveys of persons handling dromedary camels in this region could help to determine whether silent or unrecognized infections are being maintained in humans.

Technical Appendix Dromedary camel population densities in 3 regions in Kenya during 2 periods and average numbers of dromedary camels in Kenya, 1992–2013.
