# Supplementary material for: Antibodies against MERS Coronavirus in Dromedary Camels, Kenya, 1992–2013
Source: Emerg Infect Dis. 2014 Aug;20(8):1319–22. doi: 10.3201/eid2008.140596 (PMC4111164; doi:10.3201/eid2008.140596)
Supplement: Technical Appendix — Dromedary camel population densities in 3 regions in Kenya during 2 periods and average numbers of dromedary camels in Kenya, 1992–2013. [file 14-0596-Techapp-s1.pdf]

Article DOI: http://dx.doi.org/10.3201/eid2008.140596

Antibodies against MERS Coronavirus in Dromedary Camels, Kenya, 1992–2013

Technical Appendix

Technical Appendix Table. Dromedary camel population densities in 3 regions in Kenya during 2 periods*

Region	Area, km^2	No. camels during 1991–2000	Camel density during 1991–2000, no./km^2	No. camels during 2000–2013	Came density during 2000–2013, no./km^2
Rift Valley					
Baringo	11,075	3,280	0.30	5,376	0.49
Kajiado	21,293	268	0.01	607	0.03
Laikipia	8,696	3,829	0.44	2,170	0.25
Narok	17,921	0	0.00	145	0.01
Samburu	20,182	15,430	0.76	20,597	1.02
Turkana	71,598	67,097	0.94	69,380	0.97
West Pokot	8,418	2,731	0.32	1,587	0.19
Total	159,183	92,635	0.58	99,862	0.6
Northeastern					
Garissa	45,720	60,498	1.32	76,953	1.68
Mandera	25,798	97,031	3.76	95,023	3.68
Wajir	55,841	172,826	3.09	192,614	3.45
Total	127,359	330,335	2.59	364,59	2.9
Eastern					
Isiolo	25,336	60,079	2.37	63,522	2.51
Kitui	24,385	3,112	0.13	14,507	0.59
Machakos	5,953	0	0.00	1,104	0.19
Marsabit	66,923	89,682	1.34	129,143	1.93
Total	122,597	89,682	0.73	208,276	1.7

*Camel density data was calculated on the basis of livestock counts conducted by the Department of Resource Surveys and Remote Sensing as part of an ongoing Kenya-wide rangeland monitoring program (*1*). Population estimates were calculate by using Jolly's method 2 and averaged for 1991–2000 and 2000–2013 to minimize the influence of stochastic variation in the survey data.

Reference

1. Ottichilo W, Grunblatt J, Said M, Wargute P. Wildlife and livestock population trends in the Kenya rangeland. In: Prins HT, Grootenhuis J, Dolan T, editors. Wildlife conservation by sustainable use. Amsterdam: Springer; 2000. p. 203–18.

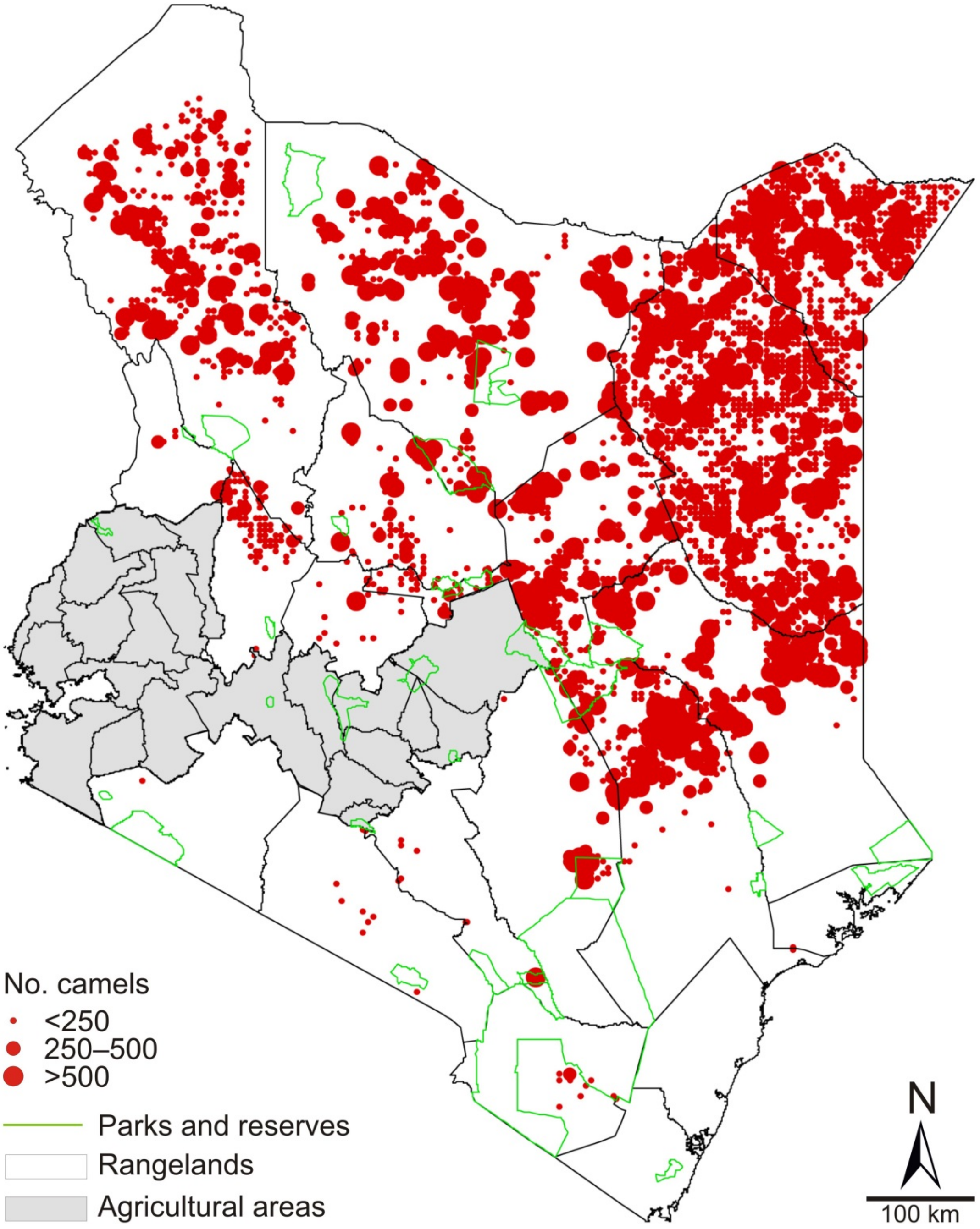

Technical Appendix Figure. Average numbers of dromedary camels in Kenya, 1992–2013.
